# Supplementary material for: Sorption Properties of PET Copolyesters and New Approach for Foaming with Filament Extrusion Additive Manufacturing
Source: Polymers (Basel). 2023 Feb 24;15(5):1138. doi: 10.3390/polym15051138 (PMC10006899; doi:10.3390/polym15051138)
Supplement: Supplementary file 1 [file polymers-15-01138-s001.zip › polymers-2219047-supplementary.pdf]

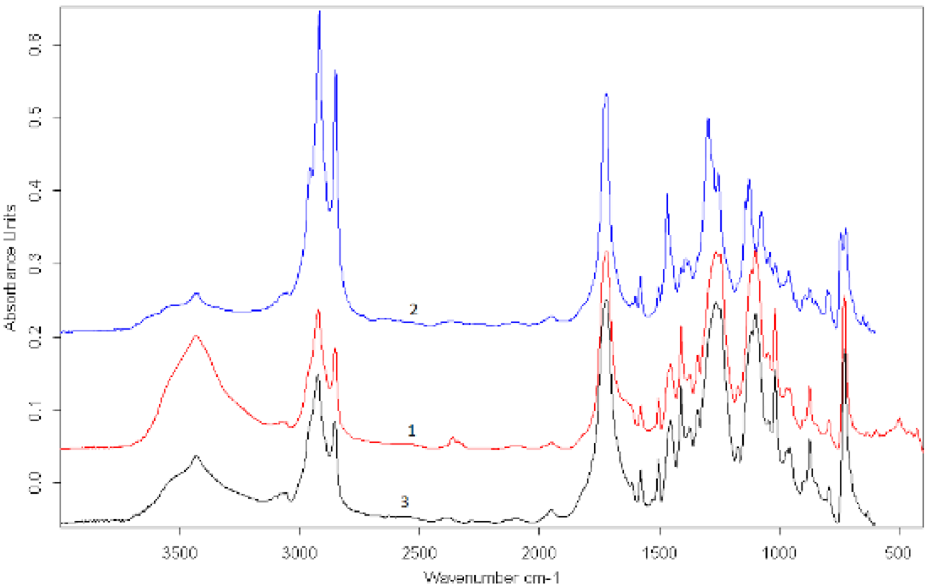

Figure S1. FTIR spectrum of PETG polyester; 1 – initial sample; 2 – sample after incomplete desorption; 3 – sample after 1 year of incomplete desorption.

Table S1. Process parameters for the production of PETG filaments

Process parameter	Value
Material drying temperature, °C	60
Material drying time, hour	8 – 10
Extruder temperature profile, °C	240-275-260-265
Spinneret forming diameter, mm	2.5
Cooling bath temperature, °C	70
Die head forming hole, mm	2.5
Spinneret drawing, %	200 – 250
Target filament diameter, mm	1.62
Ester sorption time, hour	9
Sorption temperature, °C	20
Extent of sorption, %	5.5
Filament diameter after sorption, mm	1.77

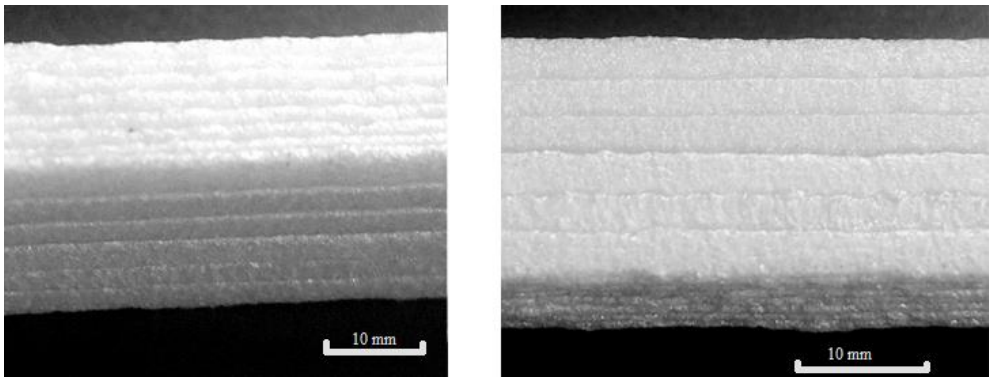

(a) (b)

Figure S2. Appearance of the surface of the 3D- printed PETG foam: a - view of the surface from above; b - view of the surface of the built platform.
